# Burns and frostbite in the Red Army during World War II

**DOI:** 10.1186/s40779-017-0114-9

**Published:** 2017-02-08

**Authors:** Vladimir Sokolov, Alexey Biryukov, Igor Chmyrev, Mikhail Tarasenko, Pavel Kabanov

**Affiliations:** Department of Thermal Injuries of the S.M. Kirov Military Medical Academy, Major of Medical Service, Zagorodnyy Prospect, 47, 190013 Saint-Petersburg, Russia

**Keywords:** Thermal injury, World War II, Statistics of burns and frostbite, Specialized burn centers

## Abstract

The start of World War II (WWII) led to the deployment of combat troops in several continents. Destruction and many casualties among both the military and civilians became an inevitable consequence. A large amount of people injured were in need of life-saving treatment and a speedy return to duty. Intensive studies of the specific issues of diagnosis and treatment of thermal injury were conducted in the Soviet Union before the war. The first special units for patients with burn injuries were created, and the first specialists received their first clinical experience. The contributions of famous Soviet scientists in the development of the treatment of burns and frostbite in WWII are studied in this article. The structure of thermal injuries among military personnel and the results of their treatment are shown. Treatment, classification and quantity frostbite in the structure of sanitary losses during the WWII are studied in this article.

## Treatment of patients with burns prior to the WWII

The Soviet Union, its allies and its opponents had no specialized medical units for patients with burn injuries in military or civilian hospitals when the WWII began [1, 2]. At the same time, the problems of pathogenesis and the surgical treatment of burns were considered at the XVI and XXIV Congresses of Surgeons of the Soviet Union (1924, 1938) and the VI Congress of the Ukraine (1936). A three-degree classification for burns was developed and functioned at that time. First-degree burns were defined by skin lesions, which were characterized by intense erythema and moderate edema of the skin. Second-degree burns were characterized by formation of thin-walled bubbles with light yellow contents in addition to the clinical features described above. Moreover, this process could occur within 2 days after exposure of the skin to high temperatures. A necrosis of tissues occurred at “a greater or lesser depth” with third-degree burns [3].

It is impossible not to note that historically unprecedented innovations - intravenous infusion systems and, in particular, blood transfusion systems- first appeared in the 1920s. These procedures were used as a means of treatment for burns. In fact, a burn treatment system did not exist at all until the 1920s. The treatment of burn wounds was understood as burn disease treatment. Principles of treatment for the burn disease, particularly infusion therapy, in the early post-traumatic period began to form in the 1930s [4]. Antishock infusion therapy consisted of small volumes (less than 1liter per day) of plasma and blood, with an emphasis on large doses of opioid analgesics. Operative restoration of the skin was limited due to lack of appropriate instrumentation. Foreign countries had some success in certain areas but still had problems in the treatment of severe burns and their complications, rehabilitation and social reintegration of patients with burn injuries [5].

A concentration of patients with burn injuries at the Institute of Emergency Medicine in Leningrad began in the mid-1930s. The experience of their treatment was the basis for I.I. Dzhanelidze to make a keynote address on the appropriateness and necessity of treatment of patients with burns in specialized hospitals at the XXIV Congress of Surgeons of the Union of Soviet Socialist Republics (USSR) in 1939. However, before the war, the supernumerary unit reduced its work.

In the structure of mortality and morbidity statistics, burns were insignificant (0.36–0.79%) in previous wars [such as the war on Khalkhin-Gol (1939) and the Soviet-Finnish conflict (1939–1940)]. Patients with burns were treated in military hospitals, which did not require the creation and deployment of specialized units. A careful analysis and generalization of experiences with burn treatment was published in the I.I. Dzhanelidze’s book *Burns and Their Treatment* in 1941. This work largely defined the tactics and methods used for treating burn patients in the Red Army during WWII.

## Treatment of patients with burns during and after the WWII

Physicians in warring countries had a new problem and task associated with the organization of treatment for patients with thermal injury and their return to the army in WWII [6]. The UK had accumulated certain experience in the treatment of burns received during the conduct of hostilities by the end of 1941. Thorough analysis of the experience led to the formulation of guidelines for a new treatment strategy. For example, in his work, Rainsford Mowlem (1941) emphasized that only “a knife or sepsis are able to determine the final outcome for patients with deep burns”. In his opinion, “the difficulty in deciding on the implementation of early operative excision of dead tissue is to determine the true depth of destruction of the skin in the first hours or day after the injury”. However, this required great skill and significant doctor experience.

One more important step was the creation of the first specialized unit in the Queen Victoria Hospital, England. There an international team of plastic surgeons, led by Archibald McIndoe, treated pilots who had received deep burns to the hands and face in the air battles of Britain [7].

The invasion of the German army into the territory of the Union of Soviet Socialist Republics (USSR) was also accompanied by an increase in thermal injury among both the military and civilians. However, the incidence of burns did not exceed 0.7% in total health losses. The largest number of burn patients were in aviation (5.8%) and the Navy (mostly on destroyers and torpedo boats, 4.2%). The prevalence of burns was as follows: first-degree (0.6%), second-degree (54.4%), third-degree (44.7%).

Burn shock was diagnosed in only 2% of patients with burn injuries. Antishock therapy included management of pain morphine [anesthesia of the burnt surface (0.25–0.50% novocaine solution], maintenance of blood pressure (ephedrine 0.4–1 ml of 5% solution as a subcutaneous and intravenous injections), treatment of dehydration and hemoconcentration (NaCl 0.9% solution 500–600 ml, 50 ml of plasma at 1% of burn area, blood transfusion to 500 ml and a continuous intravenously drip, 400 ml of glucose solutions 5% - rarely), and correction of disorders acid–base balance [acidosis (20 ml of a 30% solution of sodium thiosulphate), alkalosis (10 ml 10% calcium chloride solution)].

Local treatment included enclosure: 50.8% [bandages with iodoform, chalk, with Vaseline oil, ointments (zinc, bismuth, xeroform), 2–3 layers of paraffin-wax and gypsum bandages on limbs], semi-open: 0.7% (lotion with antiseptics), open: 26.6% [bedsteads with light bulbs, tanning substances (burnt skin treatment with 5% aqueous solution of tannin or in addition to this procedure, the wetting of a 10% solution of silver nitrate)], mixed: 21.9%. Physiotherapy equipment (quartz lamp and Sollux) was used to improve the efficiency of local treatment.

Skin grafting was rarely performed; it was performed in 19.4% cases for third-degree burns. A local anesthesia was used in 84.8% cases.

The above techniques were described by Y.Y. Dzhanelidze (1941) before the war. The results of their application were generalized in the WWII by Y.Y. Dzhanelidze and B.N. Postnikov in a separate chapter, “Treating Burns”, in the multivolume work *The Experience of Soviet Medicine in the Great Patriotic War of 1941–1945* (1951) [8].

The system of staged treatment of the wounded existed during World War II in the Red Army. The first stage - was providing of self- and mutual care directly by the wounded, his fellow soldiers or a medical orderly on the battlefield. After that, the wounded were delivered into the battalion aid stations and then into regimental medical aid stations. There, the first medical assistance was provided to them (medical triage, pain relief, correction of harnesses and bandages, oral rehydration). Wounded were then sent to the medical-sanitary battalions of divisions after this first medical assistance. Extremely urgent surgical interventions to prepare them for further evacuation were performed there. Common conditions of the wounded were also stabilized there. Then, they were evacuated to the nearest mobile field hospital of the hospital base of the army. The most difficult cases were evacuated to the hospital of the hospital base at the front, and then they were evacuated to hospitals in the internal regions of the USSR.

However, patients with burns were not always evacuated from the battlefield or the nearest frontline region to regimental medical aid stations and from there, to the medical-sanitary battalion divisions and then to the hospital. The injured sometimes passed through up to 9 intermediate stages of medical evacuation, which had not been designed to provide specialized assistance for this group of injured. This occurred in the early years of the war, during the advance of German troops in an operational-tactical situation at the front that was unfavorable for the Red Army [9–11].

The time of hospitalization was largely determined by the depth of the skin destruction. A total of 80.8% of the injured servicemen with first-degree burns were in treatment no more than 1–1.5 months, 46.7% with second-degree burns were in treatment 2 to 3 months, and 62.2% with third-degree burns were in treatment 3 to 6 months. The total mean days of treatment were as follows: all: 57.3; first-degree: 16.2; second-degree: 28.2; third-degree: 82.1.

The degree of thermal injuries affected the outcome of treatment. Of servicemen with first-degree burns, 63.4% were returned to the army in 1–3 weeks. From this group, 36.6% of the injured were deemed unfit for further military service. Treatment and subsequent passage of military medical commission took from 1 to 3 months. There was no mortality.

Of servicemen with second-degree burns, 62.0% were returned to the army in 1.0–1.5 months, 36.5% were dismissed during the period from 3 to 6 months after injury, and the mortality was 1.5%. The lethal outcomes occurred in the first 2 weeks from receipt of the thermal injury in 70.1% of cases.

Of servicemen with third-degree burns, 19.5% were returned to the army after 1 – 6 months, and 62.8% were dismissed from military service after treatment, which lasted more than 6 months. Mortality was 17.7%. Lethal outcomes occurred in the first ten days from receipt of the thermal injury in 53.6% of cases [12].

Experience in treating patients with burns accumulated during the war, and the results of the use of atomic weapons prompted I.I. Dzhanelidze to create a burn unit at the Institute of Emergency Medicine, Leningrad, in 1946. However, the unit was closed after his death in 1950.

The first beds designed for patients with burn injuries appeared at the Institute of Experimental and Clinical Surgery of the Academy of Medical Sciences of the USSR (since 1948 known as the Vishnevsky Institute of Surgery) in Moscow, in 1947. The academician G.D. Vilyavin managed the treatment of patients with burn injuries.

The widespread use of napalm in the Korean War (1950–1953) led to an increase in the frequency of burns in the structure of mortality and morbidity statistics to 25.0% [13]. It also led to the active study of the problem of burn injuries in the country and the emergence of specialized centers for patients with burn injuries. For example, S.S. Girgolav founded a burn unit at the Military Medical Academy in 1953. This unit became an independent department thanks to I.S. Kolesnikov in 1960. T.J. Aryev was apprentice of S.S. Girgolav and he was the first leader of this department. It should be emphasized that the first specialized burn units were created in hospitals, which were headed by scientists with extensive experience in military surgery (A.A. Vishnevsky, M.V. Kolokoltsev, M.I. Kuzin, B.V. Parin, B.N. Postnikov, M.I. Schreiber and many others). Undoubtedly, these great surgeons the most fully understood the relevance of the problem of burns. “Burn beds and chambers” were transformed into a burn unit in the Vishnevsky Institute of Surgery in 1960. Professor M.I. Schreiber was the first leader of this unit. Burn units were opened in Kiev in 1959, in Donetsk in 1960, and in Nizhny Novgorod in 1961 [14].

## Treatment of cold injury during the WWII

Military doctors of our country also studied cold injuries. A laboratory to study the effects of low temperatures on living organisms was created at the hospital surgical clinic of the S.M. Kirov Military Medical Academy, in 1934. S.S. Girgolav was also the leader of this laboratory. Developed by a team of specialists, a system of preventive and medical-evacuation measures was tested during the armed conflict with Finland in 1939–1940.

All information relating to mortality and morbidity statistics in the Soviet-Finnish war is extremely contradictory and incoherent. One of the main reasons for this problem is the falsification of the original data. A total of 48,745 people were killed, and 150,863 people were wounded. This was reported to the General Staff of the Red Army at the VI session of the Supreme Soviet of the USSR (March 29, 1940). No one refuted these figures for half a century.

According to statistical research that was declassified in 1993, the total number of those killed and injured from Red Army in the Soviet-Finnish war was approximately 265,000 people. Of the wounded, those contused and burned were 188,671, those suffering from frostbite were 17,867, and those that were sick were 58,370 [15]. The proportion of frostbite was 6.7% of total of sanitary loss and 9.4% of the sanitary losses of surgical profile. I.e. almost each tenth of them needed surgical care [16]. In the Finnish army, the number of wounded was 66,000 people, of whom 12% had frostbite [17].

The main achievements of Soviet military medicine of this period were the development of warning systems and the prevention of frostbite in the units of the Red Army and Navy and the creation of frostbite classifications (1940). Experience in providing care to patients with cold injuries during the fighting showed that the three degrees of classification of that time were very inconvenient, because it was based on an unfounded analogy with burns. Four degrees of classification were developed and were applied at the end of the war. This created a number of fundamentally new positions and showed its advantages in a short time. The author of this classification, T.Y. Aryev, wrote in 1942: “Classification of frostbite is possible only in a reactive period and the vast majority of frostbite captures extremities of the body of the body, mainly fingers and toes. Reactive progressive edema should be considered as the boundary of the “hidden” and “reactive” periods. First-degree frostbite is characterized as a disorder of skin blood circulation without irreversible damage (necrosis). Necrosis of the skin surface layers to the Malpighian layer is defined as second-degree frostbite. Necrosis of all layers of the skin, including the Malpighian layer and subcutaneous fat, occurs in third-degree frostbite. Necrosis of muscle and bone is typical for fourth-degree frostbite”.

In addition, the method of first aid from the early period (rapid warming in warm water) was revised and active surgical tactics for fourth-degree frostbite in hospitals were implemented.

One of the leading specialists in military surgery of the USSR, the head of the Medical Service of the Karelian Front during the WWII, I.A. Klyuss, gave the following assessment conducted by Soviet doctors’ organizational arrangements: “Creating an organization system for care and treatment of the frostbitten in the stages of medical evacuation, which is based on the classification of frostbite by T.Y. Aryev, allowed us to completely stop the evacuation of injured with first-degree frostbite from the military area. It also contributed to a complete recovery of injured with second-degree frostbite and treatment for third-degree frostbite in special units in the evacuation hospitals’ army bases, with evacuation of the injured only for fourth-degree frostbite for treatment in special hospitals at the front for the frostbitten.” [18]. It contributed to the implementation of a number of important organizational measures aimed at addressing the shortcomings in planning, logistical and medical support in the shortest time. When Germany attacked the Soviet Union in 1941, the Red Army successfully used these lessons for four years of cruel winter fights. Moreover, when our allies encountered problems with cold injury at the Western Front, they immediately sent a group of their leading experts to the USSR for a study of the national experience in the prevention and treatment of frostbite [19].

Frostbite accounted for 2.0–4.0% of the combat surgical trauma in the Red Army. Frostbite accounted for 1.0–2.0% of the total mortality and morbidity statistics in the Army and 5.4% in the Navy. Victims of cold injury were 12.5% of the total victims in the Northern Fleet compared to the Baltic Fleet (3.0%) and the Black Sea Fleet (0.7%). Marines suffered from frostbite the most often.

Most of the frostbite (95.6%) occurred in the period from November to March. One-third of the cases occurred in January. The frequency of frostbite decreased as follows: in the 1st year - 51.3% of cases, in the 2nd - 30.6% of cases, in the 3rd - 12.0% of cases, and in the 4th - 6.1% of cases. The reasons for this were the measures of collective and individual prophylaxis and the milder climate of Western Europe. Information about the structure of cold injury was not exact. I-second-degree frostbite made up 70.0–90.0% of the incoming patients for medical care. Most of the affected (83.0–91.0%) had suffered frostbite on their lower limbs. Fourth-degree frostbite was on the hands in 4.5–8.4% of the cases and on the feet in 12.3–26.4% of the cases [20].

Of the servicemen with second-degree frostbite, 100% returned to the army, 98.5% of soldiers with third-degree frostbite returned to the army, and 60.5% of soldiers with fourth-degree frostbite returned to the army, according to data from one of the evacuation hospitals. However, only 18% of servicemen with frostbite were returned to the army from a specialized hospital in Vologda in 1943.

Lethal outcomes were 0.2–0.3% of patients with fourth-degree frostbite. However, lethality for fourth-degree frostbite was 10.0% in some evacuation hospitals. This was associated with prevalence of necrosis and with incorrect surgical tactics [21].

The above facts have underlined the continuous improvement of tactics and methods of treatment of this pathology for the country in the years of the war and immediately after the victory. Several scientific and practical conferences on the problem of cold injury were conducted (Sverdlovsk, 1941; Vologda, 1944) that published monographs [22].

Our allies and enemies actively studied during the hostilities. N. Killian published his experience with treating frostbite in 1981. He led the surgical service of the 16th German Army, which surrounded Leningrad. It is known that inhuman fascist experiments on humans were conducted in the concentration camp in Dachau to study general cooling [23]. Japanese scientists engaged in experiments on Chinese, Soviet and Manchus prisoners of war in the infamous Detachment 731. In particular, they studied the endurance limits of the human body in certain conditions such as low temperature. They also conducted experiments on frostbite. Prisoners of war were forced to keep their hands and feet in special boxes with ice, until frostbite of limbs was occurred. Repeatedly these "experiments" were carried out on the street, under low environment temperatures [24]. A total of 250 medical articles on the experiences and results of the treatment of frostbite in the American and German armies in Europe from 1941 to 1945 were published in the United States during and immediately after WWII [25]. The authors noted that the methods and surgeries, which were used in the hospitals of the Wehrmacht, had been more radical and difficult than the American medical tactics. This was largely because deeper tissue injuries had been greater in the German army than in Allied troops.

## Conclusion

The Great Patriotic War contributed to the accumulation of significant experience in the treatment of thermal injury in the Soviet Union. Its comprehension and analysis led to the creation of specialized clinics and units. A similar pattern was observed in foreign countries. Modern time dictates the relevance of further improvement of the techniques and methods of treatment of patients with thermal injury. Therefore, we always have to remember the names and work of those who had done everything possible to save lives in the difficult war years. We should also think of who will save patients with thermal and frostbite injuries in difficult times.

## Abbreviations

UK: United Kingdom
USSR: Union of Soviet Socialist Republics
